# Retraction: Treatment noncompliance level among patients with type 2 diabetes mellitus: A hospital based cross-sectional study in Bangladesh

**DOI:** 10.1371/journal.pone.0287948

**Published:** 2023-07-24

**Authors:** The PLOS ONE Editors

After this article [1] was published, it came to light that the authors did not have permission to use the MMAS^®^-8 scale. This issue has not been resolved and so the *PLOS ONE* Editors retract the article. The article [1] contents were removed at the time of retraction due to the permissions issue.

The authors either did not respond to the final editorial decision or could not be reached.

Please note that the MMAS^®^-8 scale (used in this study) was originally published in 2008 [3], but the copyright for the scale was registered by Donald E. Morisky on September, 21, 2018 (U.S. Copyright Registration No. TX0008632533/2018-09-21). The MMAS^®^ trademark was also registered by Donald E. Morisky on January 29, 2019 (Reg. No. 5837374). Any reference to “MMAS” in this article [1] is for research fair use purposes.

Of note, additional issues came to light in our editorial assessment of this case that were not fully resolved prior to the retraction:

- A prior article [2] reported a study that sampled a substantially larger population to address a similar research question as in [1].
- The Methods were not reported in sufficient detail in [1], and the article did not report sufficient details as to what data were collected or how the questionnaire was validated or pretested.
- The authors stated that they did not receive ethics approval documentation for this study because it was approved during the COVID-19 pandemic. PLOS did not follow up with the institution to verify this information.
- The dataset for this study [4] includes values for the eighth question of the MMAS^®^-8 that are >1 and do not align with the reported scoring methods. The authors commented that this may reflect errors in the dataset.
